# Community capacity to acquire, assess, adapt, and apply research evidence: a survey of Ontario's HIV/AIDS sector

**DOI:** 10.1186/1748-5908-6-54

**Published:** 2011-05-28

**Authors:** Michael G Wilson, Sean B Rourke, John N Lavis, Jean Bacon, Robb Travers

**Affiliations:** 1 The Ontario HIV Treatment Network, Toronto, Canada; 2 McMaster Health Forum, Hamilton, Canada; 3 Centre for Health Economics and Policy Analysis, McMaster University, Hamilton, Canada; 4 Centre for Research on Inner City Health, St. Michael's Hospital, Toronto, Canada; 5 Department of Psychiatry, University of Toronto, Toronto, Canada; 6 Department of Clinical Epidemiology and Biostatistics, McMaster University, Hamilton, Canada; 7 Department of Political Science, McMaster University, Hamilton, Canada; 8 Department of Psychology, Wilfrid Laurier University, Waterloo, Canada; 9 Dalla Lana School of Public Health, University of Toronto, Toronto, Canada

## Abstract

**Background:**

Community-based organizations (CBOs) are important stakeholders in health systems and are increasingly called upon to use research evidence to inform their advocacy, program planning, and service delivery. To better support CBOs to find and use research evidence, we sought to assess the capacity of CBOs in the HIV/AIDS sector to acquire, assess, adapt, and apply research evidence in their work.

**Methods:**

We invited executive directors of HIV/AIDS CBOs in Ontario, Canada (n = 51) to complete the Canadian Health Services Research Foundation's "Is Research Working for You?" survey.

**Findings:**

Based on responses from 25 organizations that collectively provide services to approximately 32,000 clients per year with 290 full-time equivalent staff, we found organizational capacity to acquire, assess, adapt, and apply research evidence to be low. CBO strengths include supporting a culture that rewards flexibility and quality improvement, exchanging information within their organization, and ensuring that their decision-making processes have a place for research. However, CBO Executive Directors indicated that they lacked the skills, time, resources, incentives, and links with experts to acquire research, assess its quality and reliability, and summarize it in a user-friendly way.

**Conclusion:**

Given the limited capacity to find and use research evidence, we recommend a capacity-building strategy for HIV/AIDS CBOs that focuses on providing the tools, resources, and skills needed to more consistently acquire, assess, adapt, and apply research evidence. Such a strategy may be appropriate in other sectors and jurisdictions as well given that CBO Executive Directors in the HIV/AIDS sector in Ontario report low capacity despite being in the enviable position of having stable government infrastructure in place to support them, benefiting from long-standing investment in capacity building, and being part of an active provincial network. CBOs in other sectors and jurisdictions that have fewer supports may have comparable or lower capacity. Future research should examine a larger sample of CBO Executive Directors from a range of sectors and jurisdictions.

## Findings

Community-based organizations (CBOs) are important stakeholders in the health sector [1,2] as they not only provide a wide spectrum of programs and services to the members of their community but also play an advocacy role for broader system-level supports. As we have outlined in more detail in a previous paper [3], CBOs are typically not-for-profit organizations that: are guided by a specific mission (*i.e.*, an overall goal) shaped by commonly held values within the community they serve; have a governance structure consisting of board members elected from the members in the community; and deliver a specific set of programs or services that are shaped by the mission and values of the organization. Because they are key health system stakeholders, it is important to support their capacity to find and use relevant and high-quality research evidence. Doing so will help ensure that programs, services, and advocacy are informed by the best available evidence.

However, there are many potential challenges related to research use. Barriers that have been consistently identified across sectors include: the complexity of research evidence, organizational barriers, lack of available time, poor access to current literature, lack of timely research, lack of experience and skills for critical appraisal, unsupportive culture for research, lack of actionable messages in research reports, and limited resources for implementation [4-8]. Given these barriers, it is not surprising that a lack of uptake of research evidence has been noted in many different sectors [9-13].

While there are strategies for supporting the use of research by managers of healthcare organizations and by policy makers in government [14,15], there is still an important gap in the availability of a specific strategy for CBOs [3]. Many existing strategies for supporting the use of research evidence are based on experience and anecdotal evidence rather than on rigorous evidence of effects [8,14,16]. Moreover, strategies designed for supporting the use of research evidence by healthcare organizations and governments may not be relevant to the specific contexts and capacity of CBOs. The transferability of these strategies to CBOs is difficult to determine without first collecting evidence about their current capacity to find and use research evidence and then grappling with how to align capacity-building strategies with local realities [17-21].

In order to begin to fill this gap, we conducted an assessment of the capacity of HIV/AIDS CBOs in one Canadian jurisdiction (Ontario). The role of CBOs is particularly important for addressing the HIV/AIDS epidemic in Ontario as approximately half of all people living with HIV/AIDS in Canada reside in that province [22,23]. The province has a strong network of HIV/AIDS CBOs to respond to the local HIV epidemic, with 51 organizations serving 19 different regions at the time of this study (there are now 46 organizations) [24]. As such, this network provides essential on-the-ground support for people living with HIV/AIDS, especially for the most vulnerable [25].

We invited Executive Directors of HIV/AIDS CBOs that are funded by the AIDS Bureau of the Ontario Ministry of Health and Long-Term Care and members of the Ontario AIDS Network (n = 51) to complete an online survey. We asked each Executive Director to work with one or more managers and front-line staff to complete the survey, allowing them to respond to areas that they are most knowledgeable about but also be informed by the specific knowledge of other managers and staff.

The CBO Executive Directors in Ontario were invited to complete an adapted version of the Canadian Health Services Research Foundation's "Is Research Working for You?" survey, which has been previously validated [26,27]. The survey is organized into four areas of assessment: (1) **Acquire**: Can your organization find and obtain the research findings it needs?; (2) **Assess**: Can your organization assess research findings to ensure they are reliable, relevant, and applicable to you?; (3) **Adapt**: Can your organization present the research to decision makers in a useful way?; and (4) **Apply**: Are there skills, structures, processes, and a culture in your organization to promote and use research findings in decision making [27]? Each domain is then broken down into subsections that ask how well their organization performs specific tasks, and each question uses a five-point Likert scale (question anchors are 1 = Don't do, 2 = Do poorly, 3 = Do inconsistently, 4 = Do with some consistency, and 5 = Do well).

We analyzed the results of the survey by calculating response frequencies for each question. We did not conduct statistical comparisons between organizations due to the small sample available for the survey. Ethics approval was obtained from the University of Toronto to complete this research.

Executive Directors from 25 organizations completed the survey (response rate = 49%). These organizations collectively provide services to approximately 32,000 clients per year with 290 full-time equivalent staff. Participant/organizational characteristics are summarized in Table 1, and the results for each question are presented in Tables 2, 3, 4 to 5. The survey respondents were predominantly from organizations with an exclusive focus on HIV/AIDS. In addition, the sample appears balanced based on region served (approximately half were based in the greater Toronto area) and includes organizations with varying scale (as measured by the number of full-time equivalent staff) and service volume (as measured by the number of clients served each year). Lastly, the organizations included in our sample provide HIV/AIDS services to a broad array of populations, including women, youth, men who have sex with men, injection drug users, specific ethno-racial communities, as well as people living with HIV/AIDS.

**Table 1:** Participant characteristics

Characteristic	n (%)
**Organization type**	
AIDS service organization	23 (92%)
Organization with a mandate beyond HIV	2 (8%)

**Region**	
GTA	13 (52%)
Non-GTA	12 (48%)

**Number of FTE staff (organizational capacity)**	
<5	6 (24%)
5-10	10 (40%)
>10	9 (36%)

**Number of clients served (service demand)**	
0-250	11 (44%)
251-750	8 (28%)
>750	6 (24%)

**Ratio of FTEs to clients served**	
0-50	12 (44%)
51-75	7 (28%)
>76	6 (24%)

**Populations served**^**a**^	
People living with HIV/AIDS	25 (100%)
Women	22 (88%)
Youth	21 (84%)
Men who have sex with men	21 (84%)
Injection drug users	17 (68%)
Specific ethno-racial communities	18 (72%)
Other^b^	6 (24%)

^a^Response options were not mutually exclusive; ^b^Other populations provided by respondents were homeless/drop-ins, substance users other than injection drug users, prisoners and ex-prisoners, transient persons, Aboriginal communities, and transgendered populations.

GTA = greater Toronto area; FTE = full-time equivalent.

**Table 2:** Capacity to acquire research evidence*

Domain subsection	Factors considered	Don't do	Do poorly	Do inconsis-tently	Do with some consistency	Do well	No answer
I. Are we able to acquire research?	1. Skilled staff for research	2 (8%)	7 (28%)	7 (28%)	4 (16%)	1 (4%)	4 (16%)
	2. Time for research	5 (20%)	9 (36%)	6 (30%)	0 (0%)	0 (0%)	5 (20%)
	3. Incentives for acquiring research	3 (12%)	5 (20%)	7 (28%)	7 (28%)	1 (4%)	2 (8%)
	4. Resources to acquire research	7 (28%)	6 (24%)	5 (20%)	2 (8%)	0 (0%)	5 (20%)
	5. Links with experts who search for research, monitor research, or do research	8 (32%)	4 (16%)	7 (28%)	3 (12%)	1 (4%)	2 (8%)

II. Are we looking for research in the right places?	1. Peer-reviewed journals	3 (12%)	3 (12%)	8 (32%)	5 (20%)	2 (8%)	4 (16%)
	2. Non-journal reports/grey literature	1 (4%)	3 (12%)	7 (28%)	9 (36%)	1 (4%)	4 (16%)
	3. Databases	8 (32%)	4 (16%)	7 (28%)	3 (12%)	0 (0%)	3 (12%)
	4. Websites	0 (0%)	2 (8%)	10 (40%)	10 (40%)	0 (0%)	3 (12%)
	5. Working with researchers through formal and informal networking	5 (20%)	2 (8%)	6 (24%)	6 (24%)	2 (8%)	4 (16%)
	6. Hosting researchers	4 (16%)	4 (16%)	5 (20%)	5 (20%)	4 (16%)	3 (12%)
	7. Peer networks	0 (0%)	1 (4%)	5 (20%)	12 (48%)	4 (16%)	3 (12%)

*Results correspond to the number and percent of organizations included in the study selecting each response option.

**Table 3:** Capacity to assess research evidence

Domain subsection	Factors considered	Don't do	Do poorly	Do inconsistently	Do with some consistency	Do well	No answer
III. Can we tell if the research is reliable and of high quality?	1. Staff have critical appraisal skills for evaluating the quality of research	6 (24%)	6 (24%)	8 (32%)	4 (16%)	0 (0%)	1 (4%)
	2. Staff have critical appraisal skills for evaluating the reliability of research	8 (32%)	5 (20%)	5 (20%)	3 (12%)	1 (4%)	3 (12%)
	3. Links with external experts who use critical appraisal skills and tools to evaluate the quality and reliability of research	7 (28%)	6 (24%)	4 (16%)	3 (12%)	2 (8%)	3 (12%)

IV. Can we tell if the research is relevant and applicable?	1. Ability of staff to relate research to their organization	2 (8%)	6 (24%)	8 (32%)	6 (24%)	2 (8%)	1 (4%)
	2. Links with external experts to help determine whether research is relevant to the organization	8 (32%)	7 (28%)	5 (20%)	2 (8%)	0 (0%)	3 (12%)

*Results correspond to the number and percent of organizations included in the study selecting each response option.

**Table 4:** Capacity to adapt research evidence

Domain subsection	Factors considered	Don't do	Do poorly	Do inconsis-tently	Do with some consistency	Do well	No answer
V. Can we summarize the results in a user-friendly way?	Enough skilled staff with time, incentive, and resources to:						
	1. present research results concisely and in accessible language	7 (28%)	7 (28%)	6 (24%)	2 (8%)	1 (4%)	2 (8%)
	2. synthesize in one document all relevant research	7 (28%)	7 (28%)	3 (12%)	1 (4%)	1 (4%)	6 (24%)
	3. link research results to key issues facing decision makers	5 (20%)	5 (20%)	4 (16%)	5 (20%)	0 (0%)	6 (24%)
	4. provide recommended actions to decision makers	3 (12%)	6 (24%)	6 (24%)	4 (16%)	1 (4%)	5 (20%)
		
	Arrangements with external experts to:						
	5. present research results concisely and in accessible language	6 (24%)	7 (28%)	5 (20%)	1 (4%)	1 (4%)	5 (20%)
	6. synthesize in one document all relevant research	8 (32%)	6 (24%)	6 (24%)	0 (0%)	2 (8%)	3 (12%)
	7. link research results to key issues facing decision makers	6 (24%)	5 (20%)	5 (20%)	2 (8%)	1 (4%)	6 (24%)
	8. provide recommended actions to decision makers	7 (28%)	4 (16%)	6 (24%)	1 (4%)	2 (8%)	5 (20%)

*Results correspond to the number and percent of organizations included in the study selecting each response option.

**Table 5:** Capacity to apply research evidence

Domain subsection	Factors considered	Don't do	Do poorly	Do inconsis-tently	Do with some consistency	Do well	No answer
VI. Do we lead by example and show how we value research use?	1. Making research an organizational priority	2 (8%)	8 (32%)	4 (16%)	6 (24%)	2 (8%)	2 (12%)
	2. Providing resources to ensure research is acquired, adapted, and applied	9 (36%)	3 (12%)	4 (16%)	3 (12%)	1 (4%)	5 (20%)
	3. Involving staff in discussions about how research relates to the organization's goals	3 (12%)	6 (24%)	2 (8%)	9 (36%)	1 (4%)	4 (16%)
	4. Clear communication of organizational strategy and priorities	4 (16%)	1 (4%)	8 (32%)	6 (24%)	2 (8%)	4 (16%)
	5. Organizational communication to ensure information is exchanged	1 (4%)	0 (0%)	5 (20%)	10 (40%)	4 (16%)	5 (20%)
	6. Corporate culture supportive of research use	1 (4%)	1 (4%)	3 (12%)	9 (36%)	7 (28%)	4 (16%)

VII. Do our decision-making processes have a place for research?	1. Allocating time to identify researchable questions and consider research results	4 (16%)	3 (12%)	5 (20%)	9 (36%)	0 (0%)	4 (16%)
	2. Executive director/management have expertise to evaluate feasibility of options	0 (0%)	3 (12%)	4 (16%)	9 (36%)	5 (20%)	4 (16%)
	3. Consideration given to recommendations from staff who have developed or identified high-quality and relevant research	1 (4%)	4 (16%)	4 (16%)	7 (28%)	6 (24%)	3 (12%)
	4. Staff/stakeholders know when major decisions will be made	0 (0%)	3 (12%)	5 (20%)	8 (32%)	5 (20%)	4 (16%)
	5. Staff/stakeholders know how and when to contribute evidence and how it will be used	1 (4%)	2 (8%)	4 (16%)	8 (32%)	4 (16%)	6 (24%)
	6. Staff who provide evidence or analysis usually participate in decision-making discussions	0 (0%)	1 (4%)	4 (16%)	5 (20%)	9 (36%)	6 (24%)
	7. Relevant on-staff researchers are part of decision-making discussions	9 (36%)	3 (12%)	1 (4%)	6 (24%)	1 (4%)	5 (20%)
	8. Staff/stakeholders receive feedback about decisions with rationale for those decisions	0 (0%)	1 (4%)	3 (12%)	10 (40%)	5 (20%)	6 (24%)
	9. Staff/stakeholders are informed of how the available evidence informed decisions	0 (0%)	2 (8%)	4 (16%)	8 (32%)	5 (20%)	6 (24%)

*Results correspond to the number and percent of organizations included in the study selecting each response option

Overall, we found organizational capacity to acquire, assess, adapt, and apply research evidence to be low, with a couple of notable exceptions where the majority of organizations provided ratings of "Do with some consistency" or "Do well." Capacity for applying research evidence appears to be relatively strong, with approximately half of organizations indicating they "Do with some consistency" or "Do well" on all but two questions (related to time and engaging on-staff researchers) for the subdomain related to whether their decision-making processes have a place for research (Table 5, subsection VII). In addition, most CBOs indicated that they have a corporate culture that is supportive of research use (64% selected "Do with some consistency" or "Do well").

Few organizations rated any of the questions related to acquiring, assessing, or adapting research evidence higher than "Inconsistent." Areas of relative strength include identifying research evidence through peer networks, "grey" literature reports, and through websites. Capacity was lowest for the domains related to: acquiring research (subsection I); assessing the reliability, quality, relevance, and applicability of research evidence (subsections III and IV); and summarizing results in a user-friendly way (subsection V). A higher proportion of organizations selected the "Don't do" or "Do poorly" response options in these domains. Overall, the results indicate that CBOs are not only limited in skilled staff with time, incentive, and resources for summarizing results in a user-friendly way and assessing the quality and reliability of research, but they also have limited arrangements with external experts to help them.

This study gives initial insight into the capacity of CBOs to acquire, assess, adapt, and apply research in their organizations, which can provide a starting point for developing their capacity to find and use research evidence. Efforts to build capacity among CBOs should also draw on key facilitators for supporting the use of research evidence that have been cited for other groups of stakeholders, such as ongoing interactions between researchers, managers, and policy makers and ensuring research is available in a timely manner (*e.g.*, through searchable databases of research syntheses) [7]. In addition, many strategies to support the use of research evidence highlight the importance of clear summaries that are tailored to the specific audience and highlight the take-home messages from research [7,8,21,28,29].

The primary strength of this study is that it provides a baseline assessment of CBO capacity to acquire, assess, adapt, and apply research, which can be used for identifying areas for future capacity-building strategies. In addition, the data from this study can be used in future evaluations of organizational capacity after capacity-building initiatives have been implemented. However, the results should be interpreted with caution given the low response rate and the small sample size. In addition, the generalizability of our results to other Canadian and international jurisdictions may be limited as we only surveyed organizations in one Canadian jurisdiction.

Given the limited capacity to find and use research evidence, we recommend a capacity-building strategy for HIV/AIDS CBOs that focuses on providing the tools, resources, and skills needed to help CBOs more consistently acquire, assess, adapt, and apply research evidence. Such a strategy may also be appropriate in other sectors and jurisdictions given that CBO Executive Directors in the HIV/AIDS sector in Ontario report low capacity despite being in the enviable position of having stable government infrastructure in place to support them, benefiting from long-standing investment in capacity building, and being part of an active provincial network. CBOs in other sectors and jurisdictions that have fewer supports may have comparable or lower capacity.

Capacity building does not mean that CBOs need to do everything themselves. For instance, capacity building can focus on efforts to facilitate "pull" [28], such as developing and maintaining "one-stop shopping" websites that provide access to high-quality and relevant systematic reviews of the literature with user-friendly summaries (*e.g.*, Health Systems Evidence [http://www.healthsystemsevidence.org] for health system managers and policy makers and http://www.health-evidence.ca for public health) [30,31], and/or "rapid response" services, such as those developed through the Ontario HIV Treatment Network for HIV/AIDS CBOs [32] and the Health Technology Inquiry Service at the Canadian Agency for Drugs and Technologies in Health [33]. Additional efforts could also include facilitating access to research evidence by providing more links to external experts who can assist CBOs with identifying relevant research evidence and assessing its quality, reliability, and local applicability. Such efforts to support the use of research evidence will require in-depth consultation with CBOs to develop approaches that reflect their specific needs.

## Competing interests

Some of the organizations included in the study sample may receive funding from the Ontario HIV Treatment Network. JNL was Chair and then a member of the Board of Directors of an ASO in Ontario but he played no role in administering or completing the survey.

## Authors' contributions

MGW contributed to the conception and design of the study, the data analysis and interpretation, drafted the original manuscript, and incorporated revisions from the study team. SBR contributed to the conception and design of the study, the data analysis and interpretation, and helped draft and revise the manuscript. JNL contributed to the conception and design of the study, the data interpretation, and provided revisions to the manuscript. JB contributed to the conception and design of the study and provided revisions to the manuscript. RT contributed to the conception and design of the study and provided revisions to the manuscript. All authors read and approved the final manuscript.
